# Determination of Manganese in Serum with Zeeman Effect Graphite Furnace Atomic Absorption

**DOI:** 10.6028/jres.093.062

**Published:** 1988-06-01

**Authors:** Daniel C. Paschal, George G. Bailey

**Affiliations:** Centers for Disease Control, U.S. Public Health Service, U.S. Department of Health and Human Services, Atlanta, GA 30333

## 1. Introduction

Manganese is widely distributed in the environment, comprising about 0.1% by weight average crustal abundance [1]. Among the more important commercial uses of manganese are iron alloys, non-ferrous alloys, dry cells (as MnO_2_), oxidizers (mostly as KMnO_4_), and a large number of organomanganese compounds, notably methylcyclopentadienylmanganese tricarbonyl (MMT), used as a gasoline additive [2,3].

Under “normal” or non-occupational exposure conditions, low levels of manganese are found in serum, usually about 1 *μ*g/L or less [4]. During occupational exposure, the levels of manganese in serum or plasma are observed to rise, the manganese mainly bound to *β*-globulin or transferrin [4,5]. Manganese in blood is found mainly in the red cells, approximately 25 times higher in concentration than in serum [4]. The regulation of serum manganese levels seems to be homeostatic under “normal” conditions. Exposure to either inorganic or organic manganese has been shown to cause an increase in serum manganese [5].

Generally speaking, there are two conditions in which biological monitoring of manganese is important: 1) occupational exposure, in which serum manganese is elevated; and 2) some nutritional states in which a manganese deficiency is observed, reflected by near zero serum manganese [6].

The adverse health effects of increased manganese absorption include CNS effects, especially with organomanganese compounds. Symptoms which have been observed include dyspnea, fever, tachycardia, and Parkinsonian muscle weakness and rigidity [7]. Deficiency of manganese, on the other hand, has been suggested to be related to osteoporosis-like decalcification of bone [6].

The normal range of serum manganese values are from about 0.4 to 1.0 *μ*g/L [8–10]. Any proposed method for manganese in serum must have a detection limit commensurate with these values. Of equal importance is the avoidance of contamination by manganese, which is ubiquitous.

The method to be described is suitable for measurement of serum manganese in either nutritional or occupational exposure settings. The detection limit is about 0.2 *μ*g/L, with linearity observed up to 12 *μ*g/L. Within run precision, as calculated by analysis of variance (ANOVA), is about 10% for analysis of reference material from the U.S. National Bureau of Standards.

## 2. Experimental

### 2.1 Instrumentation

A Perkin-Elmer Zeeman/5000 atomic absorption spectrophotometer^1^ was used, equipped with an AS-40 autosampler and DS-10 Data Station (Perkin Elmer, Norwalk, CT USA). A Perkin-Elmer “Intensitron” hollow cathode lamp was used, with instrumental and spectroscopic parameters as in table 1. Pyrolytic graphite platforms and furnaces were used throughout.

### 2.2 Reagents

Serum diluent was prepared with Triton X-100 (Fisher Scientific Catalog # CS-282-4M), using 500 *μ*L diluted to 100 mL with ultrapure water. The water used was polished with a Milli-Q system (Millipore Corp.) to a resistance of 18 MΩ/cm.

### 2.3 Sample Collection

The collection of uncontaminated serum specimens is critical to the success of this determination.

Subramanian and Meranger [10] have described a procedure for serum collection which includes the use of plastic cannula to avoid manganese contamination. Veillon [11] has suggested the use of “siliconized” metal needles in which the surface has been rendered hydrophobic, thus minimizing metal contact with the specimen.

To apply this method to an osteoporosis study, a protocol was used for serum collection which is based upon our previous experience in collection of serum for determination of zinc and iron [12] and is similar to one described by Jarvisalo et al. for collection of uncontaminated whole blood for manganese determination [13]. In our protocol, two 15 mL portions of blood are collected sequentially in “standard” serum tubes using a 20 gauge multiple sampling needle (Becton-Dickinson Catalog # 5749), followed by collection of 7 mL of blood in a serum tube specially prepared to minimize trace metal contamination (Becton-Dickinson Catalog # 6526). After allowing the “trace metal” specimen to clot at room temperature, the blood was refrigerated at 4 °C and centrifuged to harvest the serum. Serum was transferred to 1.8 mL “Nunc” brand cryotubes (Catalog # 3-68632), and stored at −20 °C until analysis.

To evaluate the potential contamination from the collection and storage equipment used, a “lot testing” procedure based on statistical methods of A. Wald [14] was performed. None of the collection or storage tubes or needles (N = 10) tested had detectable manganese.

### 2.4 Contamination Control

In addition to the procedures referred to above, manganese contamination must be rigorously avoided in the specimen processing procedure itself. Glassware and plasticware were cleaned with aqueous detergent followed by rinsing with copious quantities of ultrapure water; then they were soaked overnight in 25% v/v reagent grade nitric acid. After additional rinses with ultrapure water, the plastic or glassware was then dried in a Class 100 facility, and stored in a dust-free area. Pipet tips were rinsed with ultrapure water, then with specimen or diluent before use. Linear polyethylene or polystyrene autosampler cups were cleaned as above [8,10,11].

### 2.5 Procedure

Prepare matrix modifier (0.5% v/v Triton X-100) on a weekly basis, to minimize contamination due to handling. Dilute the well-mixed serum with an equal volume of 0.5% Triton X-100, and inject 20 *μ*L aliquots via the autosampler, making duplicate area measurements. Calibration is accomplished with a curve prepared with (1+1) Triton X-100/water, using the instrumental conditions given in table 1, Peak area was used for calibration throughout.

## 3. Results and Discussion

Determination of a series of “control” sera by the proposed method gave the results in table 2. The three pools determined had nominal manganese concentrations above those expected for “normal” sera; probably as a reflection of contamination during the collection or dispensing process. Accuracy of the procedure is evidenced by both analysis of RM 8419, with a nominal value of 2.6 *μ*g/L, and by recovery estimates on “spikes” sera. Recovery of manganese to serum R576 gave an average of 102.9% for four replicate additions of 80, 160, and 240 pg manganese.

Twenty-two serum specimens were analyzed for manganese from participants in the initial phase of an osteoporosis pilot study. Results of these determinations gave a mean value of 1.3 *μ*g/L, with a range of 0.56 to 2.7 *μ*g/L. These data are quite similar to those reported by Subramanian [10], who reported data for 30 subjects. We feel that these results are indicative of a collection system that does not introduce appreciable manganese into the collected serum specimen.

**Figure 1:**
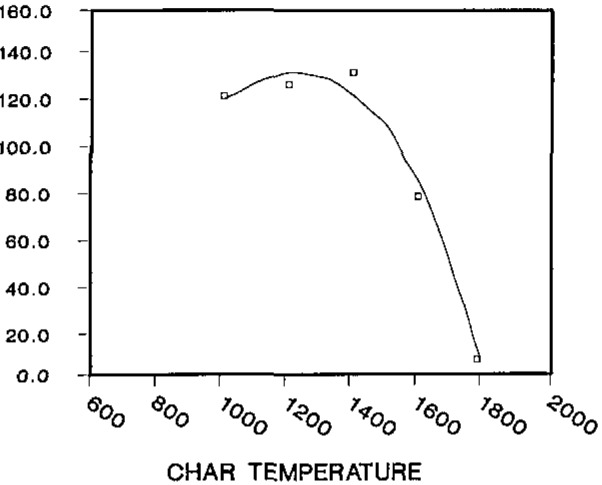
Char temperature optimization. R576 serum pool, 20 μL injection.

Optimization of the char temperature, as described by Welz [15] is shown in figure 1. The char temperature chosen was sufficient to reduce the background absorbance to approximately 0.04–0.10 A without analyte loss. The characteristic mass, pg manganese required for an average absorbance area of 0.0044 A·s, is 3.2 pg, in good agreement with published values [16,17]. Linearity was evaluated by extending the calibration curve to about 0.30 A·s, which corresponds to serum with a manganese value of 12 *μ*g/L, The calculated linear regression line gives an estimated slope of 8.57×10^−3^ A·s per *μ*g/L for both diluted serum and water (both 1 + 1 with matrix modifier).

The proposed method is rapid, convenient, and accurate. It has been shown to be suitable for evaluation of “normal” subjects for a nutritional study. Contamination, often a limiting factor with serum transition metal studies, is successfully avoided with the simplicity of the procedure and use of a single aqueous diluent.

## Figures and Tables

**Table 1:** Furnace conditions for the determination of serum manganese

Step	1 (Dry)	2 (Char)	3 (Atomize)	4 (Cool)
Temp °C	180	1400	2400	20
Ramp (s)	5	5	1	1
Hold (s)	25	15	4	4
Flow (mL/min)	300	300	0	300
Recorder, (s)			−5	
Baseline, (s)			−1	

Lamp current 30 ma, wavelength 279.5 nm, slit 0.7 (ALT). Pyrolytic platform and furnace; 20 *μ*L injected volume.

**Table 2:** Precision and accuracy study

Pool	Target value (*μ*g/L)	Found	SD (within run)	SD (among run)	SD (total)
RM 8419	2.6	2.46	0.28	0.25	0.38
R576		4.86	0.23	0.49	0.54
“Low”		2.76	0.43	0.52	0.68

## Footnotes

1 The use of trade names is for identification only and does not constitute endorsement by the U.S. Department of Health and Human Services.
